# Evaluation of Limb Load Asymmetry Using Two New Mathematical Models

**DOI:** 10.5539/gjhs.v7n2p1

**Published:** 2014-09-25

**Authors:** Senthil NS Kumar, Baharudin Omar, Leonard H. Joseph, Ohnmar Htwe, K. Jagannathan, Nor M Y Hamdan, D. Rajalakshmi

**Affiliations:** 1 Physiotherapy Program, School of Rehabilitation Sciences, Faculty of Health Sciences, University Kebangsaan Malaysia, Kuala Lumpur, Malaysia; 2 Department of Biomedical Sciences, Faculty of Health Sciences, University Kebangsaan Malaysia, Kuala Lumpur, Malaysia; 3 Faculty of Chemical Engineering, MARA University of Technology (UiTM), Shah Alam, Malaysia; 4 Department of Orthopedic and Traumatology, Faculty of Medicine, University Kebangsaan Malaysia Medical Centre, Kuala Lumpur, Malaysia; 5 Physiotherapy Program, Faculty of Health Sciences, Asia Metropolitan University, Selangor, Malaysia

**Keywords:** asymmetry, limb loading, limb load error, modified symmetry index, symmetry index, symmetry angle, symmetry ratio

## Abstract

Quantitative measurement of limb loading is important in orthopedic and neurological rehabilitation. In current practice, mathematical models such as Symmetry index (SI), Symmetry ratio (SR), and Symmetry angle (SA) are used to quantify limb loading asymmetry. Literatures have identified certain limitations with the above mathematical models. Hence this study presents two new mathematical models Modified symmetry index (MSI) and Limb loading error (LLE) that would address these limitations. Furthermore, the current mathematical models were compared against the new model with the goal of achieving a better model. This study uses hypothetical data to simulate an algorithmic preliminary computational measure to perform with all numerical possibilities of even and uneven limb loading that can occur in human legs. Descriptive statistics are used to interpret the limb loading patterns: symmetry, asymmetry and maximum asymmetry. The five mathematical models were similar in analyzing symmetry between limbs. However, for asymmetry and maximum asymmetry data, the SA and SR values do not give any meaningful interpretation, and SI gives an inflated value. The MSI and LLE are direct, easy to interpret and identify the loading patterns with the side of asymmetry. The new models are notable as they quantify the amount and side of asymmetry under different loading patterns.

## 1. Introduction

Limb loading is symmetrical, when body weight is loaded equally on both lower limbs (Adegoke, Olaniyi, & Akosile, 2012). In practice, symmetrical limb loading is encouraged as it requires less energy for functional activities such as walking and standing (Paulus & Settlage, 2011). In clinical conditions such as stroke, total knee replacement and amputation loading is asymmetrical, as one limb loads more than the other limb (Bakirhan, Angin, Karatosun, Unver, & Gunal, 2012; Agrawal, Gailey, Gaunaurd, Gailey III, & O’Toole, 2011). Clinical evaluation of limb loading is important as asymmetrical limb loading pattern alters loading on the weight-bearing joints leading to cartilage degradation and synthesis (Hurkmans, Bussmann, Benda, Verhaar, & Stam, 2003).

In orthopedic and neurological rehabilitation, asymmetrical limb loading could lead to complications (Hurkmans, Bussmann, Benda, Verhaar, & Stam, 2003). Clinicians measure limb loading pattern as a diagnostic procedure to establish therapeutic goals and intervention (Siebens, 2012; Lee, 2012). Limb loading measurement is performed using various tools and techniques such as digital weighing scale (Adegoke, Olaniyi, & Akosile, 2012) force plates and clinical examination (Hurkmans, Bussmann, Benda, Verhaar, & Stam, 2003). Force plates are considered to be the gold standard to quantify limb loading asymmetry. They require expensive instrumentation and specialized skills and hence inaccessible to most hospitals and clinics (Brennwald, Matter, & Perren, 1974; Ctercteko, Dhanendran, Hutton, & Le Quesne, 1981; Nigg, & Liu, 1999). Recent studies have used Nintendo Wii balance board (Deutsch, Borbely, Filler, Huhn, & Guarrera-Bowlby, 2008; Clark, McGough, & Paterson, 2011) and Matscan (Zammit, Menz, & Munteanu, 2010; Brenton-Rule, Mattock, Carroll, Dalbeth, Bassett, Menz, & Rome, 2012) to quantify limb loading. Other measurement tools such as the limb load monitor (Gapsis, Grabois, Borrell, Menken, & Kelly, 1982; Kathrins & O’Sullivan, 1984; Wolf, & Binder-Macleod, 1982), biofeedback system (Endicott, Roemer, Brooks, & Meisel, 1974), and ambulatory device sensors (Kljajic & Krajnik, 1987) are less explored.

Mathematical models are required to quantify limb load asymmetry regardless of the type of equipment used. The common mathematical models are symmetry index (SI), symmetry ratio (SR), and symmetry angle (SA). Robinson et al proposed the widely used SI which calculates limb load asymmetry in percentage (Robinson, Herzog, & Nigg, 1987). However, SI gives an inflated value for asymmetry (Zifchock, Davis, & Hamill, 2006). The SR involves a ratio index to evaluate asymmetry, the limitation is its low sensitivity and relatively small asymmetry value (Wall, & Turnbull, 1986). Symmetry angle (SA) incorporates a complex trigonometry formula which might be unfamiliar to clinicians (Zifchock, Davis, Higginson, & Royer, 2008). The above-mentionedlimitation justifies the need for alternative mathematical models that quantify limb load asymmetry effectively. Therefore, the first objective of this study is to present two new mathematical models Modified symmetry index (MSI) and Limb loading error (LLE) to quantify limb loading. The second objective of the study is to compare the new mathematical models against SI, SA and SR using a hypothetical data. The two models are tested in two phases of research; first phase involves developing and testing the two mathematical models based on hypothetical data. The second phase is to apply this mathematical model in the clinical population. The first phase, development and testing of the new mathematical models using a hypothetical data is presented here.

## 2. Methods

This study was conducted at the musculoskeletal research laboratory in a public university. It employs a hypothetical data for comparing different mathematical models. The data simulated in this study were systematic computational algorithms to explore all numerical possibilities of even and uneven limb loading that could hypothetically occur in human legs. The data ranged from no difference to a maximum difference, addressing all probabilities of limb loadings. Thus, the data varied between zero loading indicated by “0” to the total loading of body weight on one leg indicated by “100”.

The current mathematical models compared are: symmetry index, symmetry ratio and symmetry angle. The X right represents loading on the right limb and X left loading on the left limb.


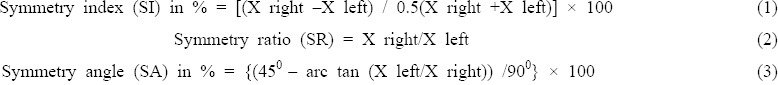


### 2.1 New Mathematical Model

The first proposed mathematical model to analyze asymmetry is Modified symmetry index (MSI). This model hypothesizes to quantify loading asymmetry in healthy and clinical population. The score of MSI is half of the symmetry index, and it has been modified for simple interpretation with reference to 100 percent. The MSI model is as follows:

Modified Symmetry index (MSI) in % = [(Affected limb loading -Unaffected limb loading) / (Body weight)] × 100

The score of MSI ranges from +100 to -100 indicating the value of asymmetry in percentage. The sign of the scores indicates the side of asymmetry. A positive sign signifies that the affected limb loads more than healthy limb, and a negative sign signifies that the affected limb loads less than the healthy limb. A score of zero indicates symmetry between limbs.

The second new mathematical model is Limb Loading Error (LLE). The Limb Loading Error model determines the loading error in reference to the expected limb loading value instead of the contra lateral limb. It also provides loading error individual to each limb. Loading error is given by the following equation.

Limb Loading Error (LLE) in % = [(Ol /Pl)-1) x 100]

were *O*_l_ is the observed loading, P_l_ is the prescribed limb loading. To apply this model in healthy population assuming symmetrical loading on limbs the value for prescribed loading is substituted by half of the body weight. A score value of zero indicates loading symmetry, and any value away from zero indicates an asymmetry in percentage. The sign of the scores indicates whether the affected limb loads more or less than expected. A positive sign indicates that the affected limb loads more than expected, and a negative sign indicates that the affected limb loads less than the expected value.

### 2.2 Procedure

Hypothetical data were used to compare the mathematical models. The hypothetical data were framed to simulate an ideal data model having variance ranging from zero to a maximum, i.e. from symmetry to maximum asymmetry. In order to simulate the data, we assumed the hypothetical body weight of an individual to be 100 kg. Using the above criterion, limb loading data points were simulated from zero to 100 kg loading on the right/left lower limbs. The generated hypothetical data identified three possible limb loading patterns; symmetry, asymmetry and maximum asymmetry. In symmetrical loading pattern the right and left limbs loads 50 kg equally. In asymmetry loading pattern, either the left or right limb loads unequal. In maximum asymmetry pattern, right or left limb takes no load or zero load, and the other limb loads the entire body weight (100 kg).

### 2.3 Data and Interpretation

Descriptive statistics are used to summarize the limb loading patterns using the five formulas. A total of 101 possible data points were derived through the hypothetical simulation. However, a total of 21 data points were extracted and presented as Table 1. The interpretation is assumed under three loading patterns: symmetry, asymmetry and maximum asymmetry. Symmetrical loading was revealed at one data point where both limb loads 50 kg equally. Two data points revealed maximum asymmetry loading pattern, with one limb loads, the total body weight and the other limb loads no load or zero load and vice versa. The remaining data points corresponded to asymmetry loading pattern. Four asymmetry data points were chosen through the systematic sampling frame method (k=N/n) to generalize the results. The first data point was chosen at random as the 10^th^ data and thereafter, every 25^th^ data was taken for interpretation.

**Table 1:** Hypothetical data simulated for a 100 kg individual considering zero loading to maximum loading on each limb

Loading at RL (Kg)	Loading at LL (Kg)	SI	SR	SA	MSI	LLE-R	LLE-L
0	100	-200	0	50	-100	-100	100
5	95	-180	0.052	-46.652	-90	-90	90
10	90	-160	0.111	-42.955	-80	-80	80
15	85	-140	0.176	-38.880	-70	-70	70
20	80	-120	0.25	-34.404	-60	-60	60
25	75	-100	0.333	-29.516	-50	-50	50
30	70	-80	0.428	-24.223	-40	-40	40
35	65	-60	0.538	-18.554	-30	-30	30
40	60	-40	0.666	-12.566	-20	-20	20
45	55	-20	0.818	-6.345	-10	-10	10
50	50	0	1	0.000	0	0	0
55	45	20	1.222	6.345	10	10	-10
60	40	40	1.5	12.566	20	20	-20
65	35	60	1.857	18.554	30	30	-30
70	30	80	2.333	24.223	40	40	-40
75	25	100	3	29.516	50	50	-50
80	20	120	4	34.404	60	60	-60
85	15	140	5.666	38.880	70	70	-70
90	10	160	9	42.955	80	80	-80
95	5	180	19	46.652	90	90	-90
100	0	200	∞	∞	100	100	-100

*Note*. RL= right limb; LL= left limb; SR= symmetry ratio; SI= symmetry index; SA= symmetry angle; MSI= modified symmetry index; LLE-R= Limb Loading error at right, LLE-L= Limb Loading error at left.

## 3. Results

### 3.1 Comparison of Symmetry Using Different Formulas

All the five formulas are able to interpret perfect symmetry between limbs (Table 1). In case of SI, SA, MSI, and LLE models, the symmetry was represented by a value of zero. However, symmetry ratio denoted symmetry as the value one.

### 3.2 Comparison of Maximum Asymmetry Data

Two data points from the hypothetical model revealed maximum asymmetry (Table 1). The first data point is indicated with the right limb loaded zero or no load and left limb loaded the total body weight (100 kgs). The data values from mathematical models are shown in Table 1 as SI (-200), SA (50), SR (0), MSI (-100), LLE (R) (-100) and LLE (L) (100). The second data point shown with the right limb loaded total body weight and left limb loaded no load or zero load. The mathematical model data values were SI (200), SA and SR (infinity value- ∞), MSI (100), LLE (R) (100) and LLE (L) (-100).

### 3.3 Comparison of Asymmetry Data 

**Figure 1:**
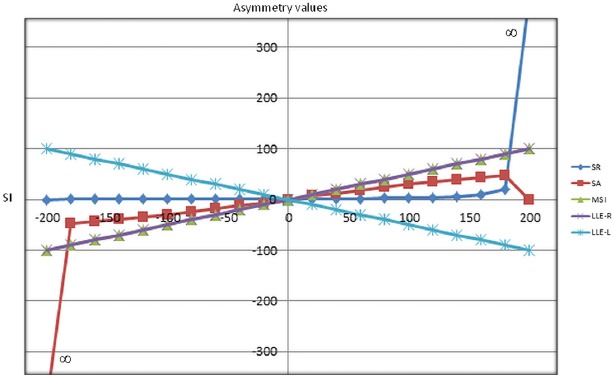
Different mathematical model representing Limb loading asymmetry values of the hypothetical data (X axis represents SI values and Y axis represents the asymmetry values; MSI and LLE (L) overlap each other)

Four data points are considered for interpretation: The first data point was taken with the right limb loading 10kg and left limb loads 90 kg. The asymmetry was identified with all models, and the values were SR (0.11), SA (42.95), SI (-160), MSI (-80), LLE (-80) and (+80) for right and left limb respectively. The second data point was taken with the right limb loading 35 kg and left limb loading 65 kg. The asymmetry values given by the different formula were SR (0.53), SA (18.55), SI (-60), MSI (-30) and LLE (R) (-30) and LLE (L) (30). The third data point was taken with the right limb loading 60 kg and left limb loading 40 kg. The asymmetry was identified with all the formula, with SR (1.5), SA (-12.56), SI (40), MSI (20) and LLE right and left were 20 and -20 respectively. The fourth data point was considered as the right limb loading 85 kg and left limb loading 15 kg. The asymmetry values obtained as SR (5.66), SA (-38.88), SI (140), MSI (70) and LLE right (70) and LLE left (-70). Graphically, all the mathematical models indicate symmetry and asymmetry loading for the induced hypothetical data. The five equations were similar in their distribution in analyzing symmetry between limbs. All models identify symmetry at point zero except SR, which indicates at point one (Figure 1). In contrast, asymmetry values are shown differently. The SR value represents asymmetry starting from zero and peaks up to infinity. The SA represents asymmetry from 50 to infinity. The other models graphically represent asymmetry as SI from -200 to +200, MSI and LLE -100 to +100.

## 4. Discussion

This study proposed two new mathematical models to evaluate limb load asymmetry and compared the new model with the existing current models. In practice limb loading measurement using mathematical models serve as an objective evaluation tool in the diagnosis of lower limb, pelvis and back injuries (Yang, 2012; Winiarski, & Czamara, 2012). Limb load measurement guides the clinician in treatment planning at different stages of injury and post-operative rehabilitation (Hurkmans, Bussmann, Benda, Verhaar, & Stam, 2003). It is important that the measurement model needs to be simple, accurate without a need for reference limb and percentage inflation. Hence this study proposes two notable mathematical models to evaluate limb load asymmetry.

The SI, SA, MSI and LLE are similar in their distribution in analyzing symmetry between limbs; with all models represent symmetry with value zero, except SR, which denoted the symmetry value as 1. Wall and Turnbull (1986) stated that the limitations to SR model, was that it failed to identify the side and extent of asymmetry. From our hypothetical data, the values of SR model are not meaningful for clinical interpretation as it ranged from relatively small to infinity values. Herzog et al. (1989) identified limitation to the SI model with the asymmetry value in ground reaction force ranged from 4% to 13, 000% during walking. Based on our hypothetical data the SI values were inflated up to 200%. The SA model incorporates a complex trigonometry formula, and it identifies both symmetry and asymmetry. However, the limitation of SA model is that the asymmetry values are not clinically meaningful, and the value varies from zero to infinity.

The two proposed mathematical models and it advantages are discussed: MSI is a good substitute for SI as it gives simple interpretation. The MSI model provides asymmetry value from zero to 100 percent, with no possible inflation. The benefits of LLE over all other models are that: Firstly, this model is applicable to individual lower limbs hence it gives an asymmetry value individual to that limb, as the current model provides one asymmetry value averaging the two limbs. Secondly, loading error, which is the difference between the observed and the prescribed limb loading value could only be explored with this model. Using this model, loading error could be studied under clinical and healthy population. In clinical conditions, like uncemented THR, fracture rehabilitation, ligament reconstruction the load to be taken on the limb is prescribed by the clinician which is to be substituted in the formula as prescribed limb loading value. For healthy population assuming equal distribution of body weight on limbs the prescribed value is substituted as half of their body weight. The clinical application and advantage of LLE are explained with the following clinical scenario:

A subject with body weight 80 kg is undergoing rehabilitation for right lower fracture. Two clinical questions are discussed. Firstly, what is the load the patient bears on his affected limb? Secondly, assuming that the patient is advised by the clinician to bear 20% of his body weight (16 kg) on his affected limb. In view of the first clinical question, the possibility of loading on the affected limb could range from zero to 100 kg. This has already been discussed using the hypothetical data. Hence the second clinical question is answered below with two scenarios:

Scenario 1 (Under loading): The subject weight bears 10 kg on his right leg (affected limb) instead of prescribed 16 kg.

Limb Loading Error (LLE) in % = [(Ol /Pl)-1) x 100]

On substitution [(10/16)-1 x 100] = -37.5

the loading error is 37.5%. The negative sign indicates that the affected limb loads 37.5% less than prescribed/expected loading.

Scenario 2 (Over loading): The subject weight bears 30 kg on his right leg (affected limb) instead of prescribed 16 kg.

[(30/16)-1 x 100]= 87.5

the loading error is 87.5 %. The positive sign indicates that affected limb loads 87.5 more than prescribed/expected loading.

Thus, Limb Loading Error model allows the clinicians to follow limb loading changes in due course or following any restorative intervention. This model may be used in wider clinical population to address the asymmetry between two sides. This study has helped to reveal some of the limitations of the commonly used mathematical models of interpreting asymmetry. It also proposes a new mathematical model MSI and LLE, which could ease interpretation and evaluates limb loading in healthy, pathological and prescribed loading conditions.

The limitation of this study is that no clinical data has been tested. Nevertheless, the limb loading patterns in real clinical data obtained from different clinical studies are within the simulated hypothetical data used in this study (Lewek & Randall, 2011; Raczkowski, Daniszewska, & Zolynski, 2010; Button, Moyle, & Davids, 2010). Hence, the researchers believe that the hypothetical data comprised all the possibility of exploring limb loading patterns. Based on the hypothetical data, the hierarchical paradigm for the mathematical models is in the order of MSI, LLE, SI, SA and SR. It is suggested that the paradigm should be followed with considerable attention, until tested with real time clinical data. However, prospective studies are in progress to test the above mathematical models on different clinical populations.

## 5. Conclusions

No current mathematical model demonstrated advantage in terms of clinically meaningful quantification of loading and identification of the side of asymmetry. The MSI provides ease of interpretation and LLE estimate loading error of individual limbs under different loading patterns and conditions quantifying changes in limb loading.
